# Effects of Circuit Training According to the Feedback Type on Psychological and Physical Health of Workers with Social Anxiety Disorder

**Published:** 2018-07

**Authors:** Yeon-Ji SHIN, Joon-Hee LEE

**Affiliations:** Dept. of Sports Coaching, College of Physical Education, Kyung-hee University, Yongin, Korea

**Keywords:** Social anxiety disorder, Circuit training, Health

## Abstract

**Background:**

The effects of circuit training was investigated according to the feedback type on the psychological (social anxiety, anxiety, positive emotion) health and physical (body composition, physical fitness) health of social anxiety disorder workers.

**Methods:**

Sixty male workers in H Company in Seoul, South Korea with social anxiety disorder were divided into four groups (positive, negative, mixed, no feedback) by conducting a circuit training program in sport center of H company during 3 times per week, total 8 weeks from Feb 1st to Mar 31st, 2017. The results of the pre-test and post-test were analyzed as follows.

**Results:**

1) In effect of social anxiety, there were significant differences in the positive, negative, and mixed feedback groups. 2) In the effect of anxiety, there were significant differences in the positive, negative, and mixed feedback groups. 3) In the effect of positive emotion, there were significant differences in the positive, negative, mixed, and no feedback groups. 4) In the effect of body composition, body fat mass and body fat percentage have significant differences in the positive, negative, mixed, and no feedback groups. And fat free mass has significant differences in the positive and mixed feedback groups. 5) In the effect of physical fitness, grip strength, wall squat, bending forward have significant differences in positive, negative, mixed, and no feedback groups.

**Conclusion:**

The circuit training program improves the social anxiety disorder and positively affects the psychological health and physical health of participants. To participate in continuous exercise, personal training should be accompanied by the correct feedback of the leader.

## Introduction

Among OECD member countries, Korea, Mexico, and Greece have the highest annual working hours of more than 2,000 hours, of which Korea is ranked second, and national happiness is also 33rd among 34 countries (1). In Korea, employees choose their major area at the end of study and engage in economic activities as members of society (2), usually continuing to work until retirement. Modern society has become increasingly convenient with automation, mechanization, etc. However, various and complicated changes from this have resulted in excessive work for people and weakened their physical and mental health. Employees are more depressed, anxious, and stressed when exposed to job instability, heavy workload, and dangerous social psychological factors (3) that negatively impact the performance and safety of individuals and organizations, resulting in symptoms such as fatigue and loss of concentration (4). Particularly, interpersonal conflict is one of the main stress factors. Although the Korean society has increased in economic size and stressed the importance of communication, however, considering the aspect of human relations, pre-modern factors remain (5), that lead to social unrest closely related to interpersonal relations.

This social anxiety is a persistent irrational fear of being exposed to situations that others can look at, a desire for avoidance that cannot be suppressed, and a fear of acting in an embarrassing manner (6). Social anxiety disorder is a common psychiatric problem that affects approximately 7–13% of the U.S. population once in a lifetime (7, 8). In cross-cultural comparative studies, social anxiety level of Oriental countries is relatively higher than in Western countries (9, 10). In the Asian regions, also, many people have social anxiety symptoms (11). Furthermore, given that the core of social anxiety disorder is the anxiety about how others see themselves, it is assumed to be relatively more in a collectivistic Oriental culture than in an individualistic Western culture (12).

Although many previous studies have reported positive effects on mental health as treatment of treating anxiety disorders (13–16), the initial rate of adults that begin an exercise program is up 50% (17). It is reported that 59% of the adults dropped out diagnosed with depression and prescribed exercise and anti-depressants for 4 months (18). Though the positive effect of exercise is proven in anxiety disorder, but it is difficult to continue. Three major factors influencing exercise adherence are psychological status, physical characteristics, and sociodemographic background (19–21). In the psychological state, the kind of feedback given by the leader will affect the exercise adherence and the feedback is the important variable for the exercise adherence of the participant through several previous studies (22–24).

This previous studies related to feedback on exercise learning have focused on improving the technical skills by acquiring exercise skills. However, participants in daily life sports participate to reduce the daily life stress and anxiety and strive for psychological stability. Therefore, it is necessary to provide adequate linguistic feedback, including positive and negative feedback effective in (22, 23) interest, pleasure (24), tension relaxation and sustaining exercise adherence. Researchers in management of organizational behavior also argue that positive and negative feedback should be provided separately to increase the effectiveness of feedback. In other areas, combinations of positive and negative feedback should be used, considering the side effects of negative feedback (25). Therefore, the treatment of mixed feedback except the single application of positive and negative feedback is also worthy of verifying the effectiveness of the social anxiety reduction as a method of continuing participation in the exercise.

The purpose of this study was to investigate whether social anxiety was changed by the effect of circuit training program according to feedback type as well as measurement of social anxiety disorder of employees. This approach may reveal the improvement of social anxiety disorder through circuit training program also the feedback type as an important variable in psychological health, physical health and participation in continuous exercise.

## Methods

### Research Subject

Subjects in this study were recruited male office workers in their 30s and 40s in H Company in Seoul, South Korea in 2017. Subjects visited the sports center in the workplace and filled in a 20-item social phobia scale. Afterwards, we adopted the criteria (26) of Brown et al. (27) and Heimberg et al. (28) and assumed more than 24 points as clinically high social anxiety. The final 60 respondents were selected from a total of 196 questionnaires. In the circuit training programs of the social anxiety group, it is constructed that a total of 4 groups as the positive feedback, negative feedback, positive + negative feedback (same as above mixed feedback) and control group with no feedback (same as above no feedback) and each group is assigned to 15 subjects. During 3 times per week, total 8 weeks participation period, 0, 6, 2, and 4 participants dropped out, respectively. The specific characteristics of the subjects are shown in Table 1, and there was no significant difference between the groups in the pre-test.

**Table 1:** Physical characteristics of subjects

***Variable***	***PF (n=15)***	***NF (n=9)***	***MF (n=13)***	***NF (n=11)***	***P***
Age (yr)	35.13±2.97	36.22±4.47	36.23±4.02	35.45±3.83	.845
Height (cm)	175.36±4.58	175.84±6.87	176.58±6.01	174.60±3.25	.825
Weight (kg)	75.08±7.96	74.36±5.99	77.44±9.22	74.59±8.12	.774
BMI (kg · m^2^)	24.44±2.74	24.09±2.16	24.88±3.14	24.43±2.12	.918
Social Anxiety Score	27.27±2.81	27.11±2.42	26.08±3.43	27.00±1.84	.688
Dropout rate	0%	40%	13%	27%	

PF: Positive Feedback, NF: Negative Feedback, MF: Mixed Feedback, NF: No Feedback

### Measurement

The Social Phobia Scale (SPS) was developed already (29) and we used the Korean version of the SPS adapted already (30) assessed on a 5-point scale with a total of 20 items, and the internal consistency of the previous study was .92. The Beck Anxiety Inventory (BAI) was developed by Beck et al. (31) and we used the Korean version of BAI (32) assessed on a 4-point scale with a total of 21 items and the internal consistency was .88. The positive emotional scale was developed by Watson et al. (33) and we used questionnaires adapted by Lee et al. (34), assessed on a 5-point scale with a total of 10 items and the internal consistency was .88.

### Circuit Training Program

The circuit training program in this study was constructed based on the exercise program applied already (35, 36). As social anxiety workers, the subjects of this study tended to be afraid of others’ evaluation of them (37) (Table 2).

**Table 2:** Circuit training program

***Program***	***Type***	***Intensity***	***Time***	
Pre-exercise	Stretching	RPE 7∼11	5 mins	
Exercise	①Push-up	RPE 12∼14	18 times	15 min/2 sets
	②Stick bent over row		18 times	
	③Squat		18 times	
	④Support pull-up		18 times	
	⑤Stick military press		18 times	
	⑥Hip hinging deadlift		18 times	
	⑦Superman		18 times	
	⑧Normal crunch		18 times	
	⑨Stick lunge		18 times	
	⑩Rotation		18 times	
Post-exercise		RPE 7∼11	50 mins	

### Type of Feedback

Positive Feedback: During the exercise program, the leader emphasized the positive aspect of performance and said, “This movement is correctly performed.”, “Please continue it like that.”, and “Very good. Good work.”, and did not mention the insufficient move but provided positive results focused on achievements.

Negative Feedback : During the exercise program, the leader emphasized the negative aspect of performance and said, “This movement is incorrectly performed.”, “The movements to be corrected are like this.”, “You need to work out harder and concentrate.”, and did not mention the sufficient move but provided negative results focused on non-achievement.

Mixed Feedback: During the exercise program, the leader referred to positive and negative aspects of performance and gave feedback such as “This movement is correct and incorrect in this way.”. He applauded and pushed for continued efforts in well performed movement, and pointed out the insufficient movement and requested constant revision, and provided information related to achievement and non-achievement record continuously.

## Results

### The effect of circuit training on social anxiety according to feedback type

The main effect of group and test was revealed, and the interaction effect between group and test appeared. Social anxiety significantly decreased in all groups except negative feedback, and the positive feedback group showed the greatest decrease (Table 3).

**Table 3:** The effect of circuit training on social anxiety according to feedback type (Mean±SD)

***Variable***	***Group***	***Test***		***Δ%***		***P***	
		**Pre**	**Post**				
Social Anxiety	PF	2.36±0.14	1.67±0.14	−29.25	***	Group	.001
	NF	2.34±0.12	2.34±0.22	−0.44		Test	.001
	MF	2.29±0.17	1.94±0.08	−15.19	***	Group×Test	.001
	NF	2.40±0.17	2.20±0.10	−9.25	**		

PF: Positive Feedback, NF: Negative Feedback, MF: Mixed Feedback, NF: No Feedback

### The effect of circuit training on anxiety according to feedback type

The main effect of group and test was revealed, and the interaction effect between group and test appeared. Social anxiety significantly decreased in all groups except negative feedback, and the positive feedback group showed the greatest decrease (Table 4).

**Table 4:** The effect of circuit training on anxiety according to feedback type (Mean±SD)

***Variable***	***Group***	***Test***		***Δ%***		***P***	
		**Pre**	**Post**				
Anxiety	PF	2.58±0.37	1.47±0.30	−43.10	***	Group	.016
	NF	2.45±0.19	2.34±0.22	−4.54		Test	.001
	MF	2.48±0.37	1.79±0.28	−27.77	***	Group×Test	.001
	NF	2.29±0.30	2.06±0.20	−10.00	*		

PF: Positive Feedback, NF: Negative Feedback, MF: Mixed Feedback, NF: No Feedback

### The effect of circuit training on positive emotion according to feedback type

The main effect of group and test was revealed, and the interaction effect between group and test appeared. Positive emotion significantly increased in all groups (Table 5).

**Table 5:** The effect of circuit training on positive emotion according to feedback type (Mean±SD)

***Variable***	***Group***	***Test***		***Δ%***		***P***	
		**Pre**	**Post**				
Positive Emotion	PF	1.83±0.62	2.97±0.63	62.18	***	Group	.178
	NF	1.83±0.33	2.63±0.39	43.64	***	Test	.001
	MF	1.64±0.37	2.47±0.50	50.70	***	Group×Test	.275
	NF	1.89±0.54	2.83±0.35	49.52	***		

PF: Positive Feedback, NF: Negative Feedback, MF: Mixed Feedback, NF: No Feedback

### The effect of circuit training on body composition according to feedback type

Body fat mass, body fat percentage and fat free mass showed the main effect of test, and body fat mass and body fat percentage significantly decreased in all groups. In addition, fat free mass significantly increased in positive feedback and mixed feedback (Table 6).

**Table 6:** The effect of circuit training on body composition according to feedback type (Mean±SD)

***Variable***	***Group***	***Test***		***Δ%***		***P***	
		**Pre**	**Post**				
Weight (kg)	PF	75.09±7.96	75.09±7.67	0.01		Group	.770
	NF	74.36±5.99	74.21±5.49	−0.19		Test	.942
	MF	77.44±9.22	77.41±9.09	−0.04		Group×Test	.860
	NF	74.59±8.12	74.80±7.95	0.28			
BMI (kg/m^2^)	PF	24.44±2.74	24.43±2.58	−0.01		Group	.911
	NF	24.09±2.16	24.04±1.96	−0.21		Test	.954
	MF	24.88±3.14	24.87±3.10	−0.04		Group×Test	.818
	NF	24.43±2.12	24.50±2.12	0.30			
Body fat mass (kg)	PF	15.85±5.40	15.32±5.28	−3.36	***	Group	.673
	NF	15.22±4.29	14.57±4.13	−4.31	**	Test	.001
	MF	17.85±5.59	17.16±5.59	−3.88	*	Group×Test	.480
	NF	16.12±5.55	15.77±5.49	−2.14	*		
Body fat percentage (%)	PF	20.79±5.34	20.10±5.35	−3.31	***	Group	.716
	NF	20.26±4.23	19.43±4.21	−4.07	***	Test	.001
	MF	22.66±4.88	21.78±5.01	−3.90	**	Group×Test	.463
	NF	21.30±5.80	20.80±5.74	−2.38	**		
Fat free mass (kg)	PF	33.47±3.01	34.01±2.99	1.61	**	Group	.976
	NF	33.52±2.12	34.03±1.93	1.52		Test	.001
	MF	33.45±2.98	34.12±3.01	1.98	***	Group×Test	.809
	NF	33.14±3.10	33.51±3.12	1.12			

PF: Positive Feedback, NF: Negative Feedback, MF: Mixed Feedback, NF: No Feedback

### The effect of circuit training on physical fitness according to feedback type

All variable showed the main effect of test, and wall squat showed the interaction effect of test and group. In addition, all factors of physical strength, such as grip strength, wall squat, and bending forward, significantly increased in all groups (Table 7).

**Table 7:** The effect of circuit training on physical fitness according to feedback type (Mean±SD)

***Variable***	***Group***	***Test***		***Δ%***		***P***	
		**Pre**	**Post**				
Grip strength (Left)	PF	39.65±5.92	42.79±5.93	7.90	**	Group	.709
	NF	37.59±3.04	40.78±3.19	8.48	**	Test	.001
	MF	38.32±4.48	41.38±4.85	7.99	**	Group×Test	.558
	NF	40.05±4.59	42.05±5.20	4.99	**		
Wall squat (Sec)	PF	68.20±20.44	77.33±22.77	13.39	***	Group	.800
	NF	65.11±18.99	76.00±18.87	16.72	***	Test	.001
	MF	66.38±16.33	75.38±15.89	13.56	***	Group×Test	.048
	NF	75.36±22.62	80.82±22.46	7.24	**		
Bending forward (cm)	PF	4.46±7.71	7.70±6.27	72.65	***	Group	.861
	NF	2.61±5.95	6.22±6.11	138.30	***	Test	.001
	MF	5.15±7.88	8.19±6.92	58.96	***	Group×Test	.852
	NF	5.32±7.14	8.27±6.33	55.56	***		

PF: Positive Feedback, NF: Negative Feedback, MF: Mixed Feedback, NF: No Feedback

## Discussion

According to a study on demographic bases on the effects of regular exercise on anxiety, depression, and personality, regular exercise participation has a significant effect on anxiety reduction (38). It has positive effects on decreasing stress of participants (39). Additionally, according to the results of the study reporting that it is better to accumulate more than 150 minutes of intense physical activity every week to obtain effective health benefits for adults (40), the circuit training program of this study that can perform high intensity exercise for a short time is the most suitable exercise for the worker with time and space limitation. Moreover, visits to the sport center for exercise participation have an effect of reducing anxiety through social exposure, likely to be effective for workers frequently experiencing social anxiety. In a previous study that examined correlations between social anxiety disorder and feedback, it is difficult to conclude that positive feedback has a positive or negative impact on social anxiety disorder. Therefore, this study has the possibility of positively affecting social anxiety disorder among positive, negative, mixed, no feedback. In conclusion, positive, mixed, and no feedback were effective in decreasing social anxiety disorder and anxiety, especially positive feedback was effective. Additionally, negative feedbacks do not show significant change, so it can be predicted that this type of feedback should be avoided for the subject of social anxiety disorder. Therefore, when the leader instructs the exercise to improve the social anxiety disorder, if the feedback using the positive language is applied, the effect of the improvement of the social anxiety disorder through the exercise is likely to be high.

Seligman became aware of the neglect of the positive aspect by focusing only on the negative aspects and argued for positive psychology (41). Positive emotion has been reported to be a powerful motivational tool (42–45) for continuing the exercise through a number of prior studies, affecting the individual’s well-being and happiness (41). Because positive feedback leads to positive emotion to the learner in performing the task (46, 47), positive feedback of the leader induces positive emotions of the participants and motivation of continuous exercise, as a result, positive effect is expected to social anxiety disorder employees. This study shows that positive emotion increases significantly in all feedback groups, so that participation in the exercise program has a significant effect on positive emotion regardless of feedback type.

Body-fat reduction and fat free mass increased by conducting circuit training on 30s obese men with sedentary lifestyle (48). Applying circuit training programs with different intensity, circuit training with higher intensity was more effective in improving body composition than low intensity (49). Thus, physical fitness is closely related to body composition and physical fitness is improved by increasing fat free mass including muscle through circuit training program with resistance exercise. Conversely, although there was no difference according to the leaders’ feedback type, physical fitness results showed positive improvement, suggesting that circuit training program would be useful for improving social anxiety disorder. However, since the difference between the exercise of guidance by the learner in providing linguistic feedback and the participation in the personal exercise without feedback cannot be explained through the results of this study, it can be predicted that the circuit training program has a significant effect on improving the physical fitness of the participant.

Because of this study of each group bounce rate, the positive feedback group participated in the experiment without dropping out. The negative feedback group dropped out 6 participants (40%), the mixed feedback group was 2 (13%) and the no feedback group was 4 (27%). Among the four groups, the negative feedback group showed a bounce rate about 50%, interpreted as the same as the negative feedback group had no significant effect on social anxiety and anxiety reduction. The results of this study showed that the negative feedback group had a higher dropout rate than the leader that did not provide feedback, inhibiting the motivation of participants. To improve the social anxiety disorder, it is necessary to participate in the exercise continuously. However, it can be interpreted that the exercise instruction method through the negative language of the leader is a major obstacle for the participants to continue the exercise. Therefore, for participants to exercise continuously and improve social anxiety disorder, a method of providing negative language should be avoided. The circuit training program according to feedback type has a positive effect on employees with social anxiety disorder. Especially when leaders conducted exercise instruction with positive feedback, it was most effective in improving social anxiety disorder. Considering exercise participation rate, the leaders’ negative feedback can be classified as the type of excluded guidance as a method for improving social anxiety disorder. Therefore, it should be recognized that the leaders’ feedback type is a role that should be considered for the subjects with social anxiety disorder.

## Conclusion

The circuit training program has a positive effect on improving social anxiety disorder and conclude that the leaders’ personal training and correct feedback are needed to combine for the greater effect and continuous exercise participation. In future studies, it will be necessary to further investigate that distinguish the social anxiety disorder degree according to personal interview and to observe changes through exercise program. Additionally, based on the effect of positive feedback proved by this study, if we propose a customized circuit training program to improve the social anxiety disorder and verify the effect, it can be the basic data to solve the unstable psychological state and train health body.

## Ethical considerations

Ethical issues (Including plagiarism, informed consent, misconduct, data fabrication and/or falsification, double publication and/or submission, redundancy, etc.) have been completely observed by the authors.
